# Prioritizing Seafloor Mapping for Washington’s Pacific Coast

**DOI:** 10.3390/s17040701

**Published:** 2017-03-28

**Authors:** Timothy Battista, Ken Buja, John Christensen, Jennifer Hennessey, Katrina Lassiter

**Affiliations:** 1 National Oceanic and Atmospheric Administration, National Centers for Coastal Ocean Science, Silver Spring, MD 20910, USA; Ken.Buja@noaa.gov (K.B.); John.Christensen@noaa.gov (J.C.); 2 Washington State Department of Ecology, Lacey, WA 98503, USA; Jennifer.Hennessey@ecy.wa.gov; 3 Washington State Department of Natural Resources, Olympia, WA 98504, USA; Katrina.Lassiter@dnr.wa.gov

**Keywords:** mapping, planning, remote sensing, prioritization, decision making, seafloor, Washington State

## Abstract

Remote sensing systems are critical tools used for characterizing the geological and ecological composition of the seafloor. However, creating comprehensive and detailed maps of ocean and coastal environments has been hindered by the high cost of operating ship- and aircraft-based sensors. While a number of groups (e.g., academic research, government resource management, and private sector) are engaged in or would benefit from the collection of additional seafloor mapping data, disparate priorities, dauntingly large data gaps, and insufficient funding have confounded strategic planning efforts. In this study, we addressed these challenges by implementing a quantitative, spatial process to facilitate prioritizing seafloor mapping needs in Washington State. The Washington State Prioritization Tool (WASP), a custom web-based mapping tool, was developed to solicit and analyze mapping priorities from each participating group. The process resulted in the identification of several discrete, high priority mapping hotspots. As a result, several of the areas have been or will be subsequently mapped. Furthermore, information captured during the process about the intended application of the mapping data was paramount for identifying the optimum remote sensing sensors and acquisition parameters to use during subsequent mapping surveys.

## 1. Introduction

The U.S. has the world’s largest exclusive economic zone (EEZ, 11.7 million km^2^) and within those waters has the sovereign rights for purposes of exploring, exploiting, conserving and managing natural resources [1]. As such, the U.S. has a large incentive to map and understand our oceans and coasts. However, while the enormous size of the U.S. EEZ offers bountiful ecological, recreational, cultural, and economic value vital to supporting the Nation’s economy, the sheer size of the EEZ is also a daunting challenge to map and characterize.

The importance of the U.S. EEZ is demonstrated by the economic contribution of industries dependent on or derived from the ocean or Great Lakes, known as the Ocean Economy. For example, in 2013, the U.S. ocean-based GDP (Gross Domestic Product) contributed $359 billion dollars to the total U.S. GDP [2]. Furthermore, the contribution of the ocean economy to the total U.S. GDP has been increasing (1.9% in 2010, and 2.2% in 2013) [2], with growth expected to continue in the future. However, an increasing ocean economy has contributed to burgeoning maritime usage of the U.S. coastal zone, resulting in increased multi-use conflicts. Continued growth in coastal infrastructure developments and anticipated future increases in ocean uses will further exacerbate use conflicts [3,4].

Actions to mitigate and balance competing ocean uses would benefit from improved decision-making tools and increased usage of informational sources such as seafloor mapping data products [5,6]. Marine Spatial Planning (MSP) is a process that enables integrated, forward looking, and consistent decision making on the human uses of the oceans and coasts [7]. It can improve marine resource management by planning for human uses in locations to reduce conflict and allow us to balance and maximize the social, economic, and ecological benefits we receive from ocean resources [6]. For example, within Washington State, potential competing uses include maritime shipping, recreational uses, tribal treaty fishing, commercial and recreational fishing, resource extraction, renewable energy production, military usage, and ecological resource management [8]. Each of these activities is influenced directly or indirectly by seafloor properties and features. Furthermore, there is growing public interest [9] and directed U.S. policy initiatives [10] to increase domestic renewable energy production along the Outer Continental Shelf in federal waters of the United States. As a result, Washington [11], in addition to several other coastal states (Connecticut, Rhode Island, Massachusetts, Maryland, New York, and Oregon) have enacted state-level MSP laws or developed plans to better address conflict-use issues in coastal waters.

The Marine Spatial Plan for Washington’s Pacific Coast requires mapping key physical and biological characteristics and environmentally sensitive areas, including areas with unique or sensitive species or biological communities that may require protection [11]. Furthermore, the plan is required to include recommendations for protection of unique and sensitive biota and ocean floor features within the exclusive economic zone waters [11]. However, the ability to provide sound recommendations is predicated upon having sufficient mapping information on the extent and diversity of benthic habitats to support those findings [12]. 

As of 2014, many different research groups had mapped portions of the seafloor off Washington’s coast at varied levels of detail, using various methods [13]. These mapping data can be used to understand the most basic characteristics of the seafloor such as bathymetry, bottom type, habitats, and geology across a limited geographic scope. Additionally, mapping information can help resource managers and planners understand the distribution of habitats and the potential impacts to them, and develop plans that consider this information [8]. However, Washington has lacked a central repository for accessing seafloor mapping data and derived products from various sources, which has impeded the assessment of the spatial coverage and quality of existing data [12].

Insufficient coordination among federal, state, non-governmental organizations, tribes, and academic institutions in the planning, coordination, acquisition, and data sharing of coastal mapping data is a systemic problem common to many coastal regions [5]. While a considerable number of mapping surveys have been conducted for Washington, the data is largely a patchwork of varying quality, age, resolution, spatial datums, and spatial coverage as the surveys were collected by a wide variety of groups. Moreover, in most cases, the mapping survey extents and sensor systems used were selected to meet the explicit requirements of the respective collector, rather than a broad range of beneficiaries [14]. Washington’s MSP process has attempted to overcome these obstacles through the use of planning tools which enable better mapping coordination, and therein, initiate mapping surveys that incorporate more inclusive objectives [15].

There are very real and practical challenges to conducting better state or regional mapping coordination. Diverging geographic and application priorities, funding constraints, and failure to implement National standards or protocols among groups involved in data collection is common [5]. Furthermore, many locales lack access to the necessary collection platforms, sensor technologies, and skilled remote sensing scientists to acquire the mapping information needed [16]. However, there have been several attempts to mitigate these challenges by implementing a mapping prioritization process [17,18]. The ultimate goal of the mapping prioritization is to embrace a process which includes a broad range of user groups seeking convergence on priority areas for mapping. The underlying premise for prioritization is that insufficient funding or survey time was available to map the entire Washington State study area. Hence, future efforts are predicated on the ability to demonstrate targeted areas in which additional information is needed. Additionally, users seek to document the underlying applications of those data so that the most appropriate remote sensing systems and platforms can be applied to best address the intended management issue [19]. 

While prior prioritization efforts in other locations have been successful, several improvements to the methodologies used were identified which would increase the utility of the results for decision-making. These suggestions include incorporating: (1) web-based tools to facilitate participant input; (2) procedures to ensure equal distribution and weighting of participant input; and (3) techniques to allow for fine-scale adjustment or modification of model results [17]. In this paper, we describe the utility of the improved mapping prioritization process using web-based mapping tools to address Washington State’s MSP efforts. 

## 2. Methods

**Figure 1:**
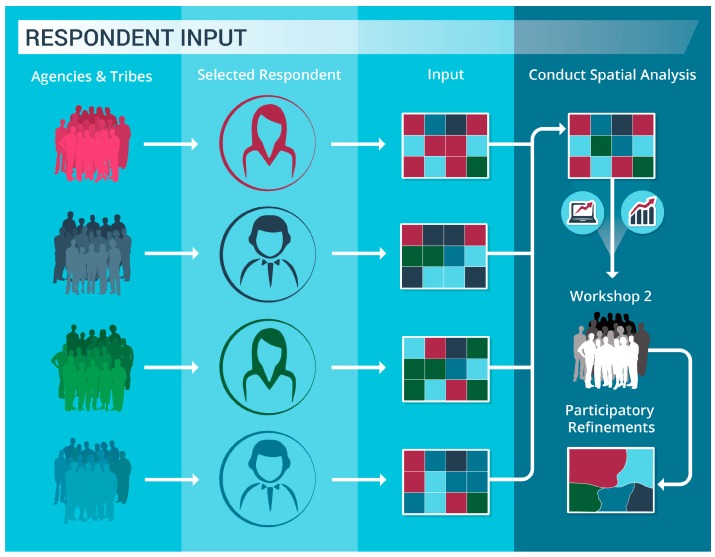
Spatial Prioritization Exercise conceptual process.

The Washington seafloor mapping prioritization process improved upon previous efforts through implementation of a web-based, Washington State Prioritization Tool (WASP). Prior to conducting the spatial prioritization exercise using WASP (described in Section 2.1), a workshop was held in Lacey, Washington (27 October 2014) to introduce participants to the concept and incorporate additional suggestions. The revised process and use of WASP hinged upon the independent participation of groups through a selected respondent (Figure 1).

Eighteen respondents, representing their respective federal and state agencies, and coastal treaty tribes (Quileute Tribe and Quinault Indian Nation), participated in the spatial prioritization exercise. Many of the respondents organized meetings with individuals in their agencies to capture and iterate entries in the WASP. Alternatively, some agencies were unable to meet collectively, but as WASP allowed the respondent to share results digitally, they were able to seek feedback from others, refine entries, and reach consensus. Each respondent completed the exercise independently using WASP to capture the mapping priorities of their respective agencies. The submitted selections were then analyzed collectively (see Section 2.2) across all the participating groups to discern patterns. The results were presented to the group during a second workshop (14 May 2015), and to achieve consensus, additional refinements were made (see Section 2.3).

### 2.1. Web-Based Prioritization Tool

The Washington State Prioritization Tool website was developed using Esri’s ArcGIS API (Application Program Interface) for JavaScript to allow invited respondents to select areas and assign priorities to the cells, justifying this priority level by choosing a management issue and up to three ranking criteria. The application contains both a query component and an edit component. The query component (“Data Layers” tab) is open to all participants while the edit component (“Prioritization” tab) is available to only invited respondents. In this case, a participant is defined as a user involved in advising selections for their respective agency, and respondent being the individual with account privileges to make selections on behalf of their respective agency. 

**Figure 2:**
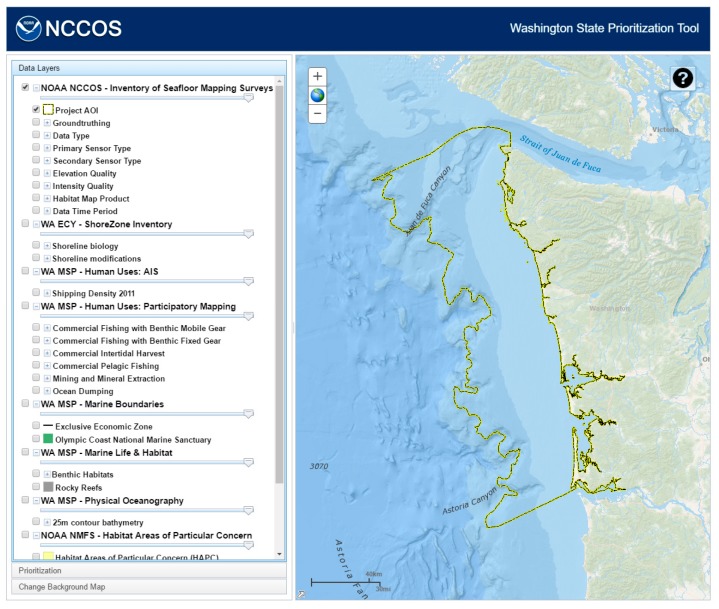
Washington State Prioritization Tool website uses map services from NOAA, Washington State, and Oregon State University.

The query component uses an interactive Table of Contents tool (a third party tool, available on ArcGIS.com), which lists all of the contents of the collected datasets in a tree-like structure. The individual layers or groups of layers can be turned on and off by clicking on the checkbox next to their names. The site uses map services from a number of different sources: NOAA (National Oceanic and Atmospheric Administration) National Centers for Coastal Ocean Science (Inventory of Seafloor Mapping Surveys), NOAA National Marine Fisheries Service (Habitat Areas of Particular Concern and Essential Fish Habitat Areas Protected from Fishing), NOAA Office of Coastal Management (Undersea Feature Place Names), Washington State Department of Ecology (ShoreZone Inventory), Washington State Department of Natural Resources (Human Uses; Marine Boundaries; Marine Life and Habitat; Kelp; and Physical Oceanography), and Oregon State University (Seafloor Mapping Data Quality; Predicted Outcrop; Physiographic Habitat; Primary Lithologic Seabed; and Seafloor Induration) (Figure 2).

**Figure 3:**
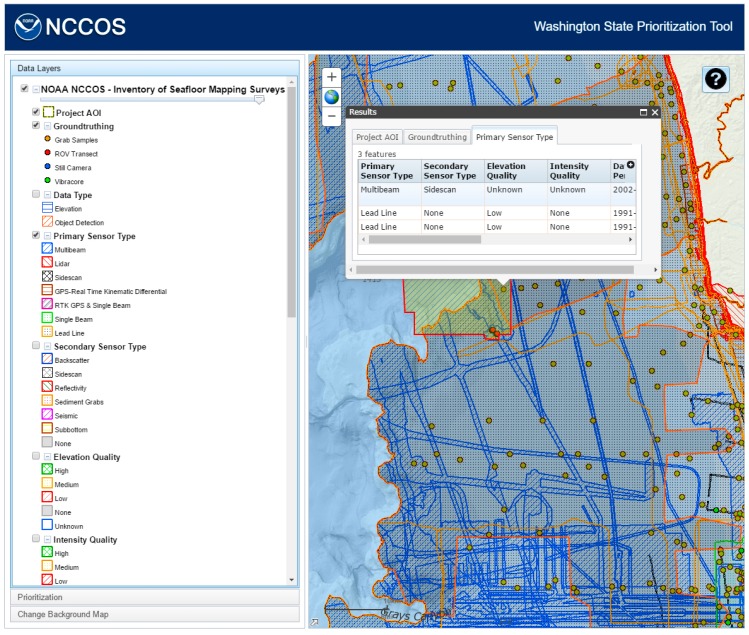
Compiled inventory of existing seafloor mapping data for Washington State made available to users through Washington State Prioritization Tool (WASP).

Additionally, the tool allowed users access, within the Table of Contents, to an inventory of existing seafloor mapping data within the study region (Figure 3). This spatial inventory of mapping data was compiled from a host of disparate sources into a standardized database. Information was gathered from the following key data holders: NOAA Office of Coast Survey, NOAA Office of Coastal Management, NOAA Olympic Coast National Marine Sanctuary, NOAA National Centers for Environmental Information, U.S. Army Corps of Engineers, Oregon State University, Washington Department of Natural Resources, Washington Department of Ecology, and several smaller groups. The data records within the viewer were organized by the extent or boundary of individual surveys, and results clipped to the edge of the project boundary (Figure 2). A host of seafloor mapping data was included in the data viewer focusing on three different categories of data: source mapping data, ground-truthing data, and derived benthic habitat map products. To improve the consistency of display and querying, the feature information collected from various sources were translated into standardized attributes and categories. Additionally, the data were reviewed and qualitatively assessed to categorize a series of attributes regarding the quality and age for existing seafloor mapping data. 

Source Data Categories:

- Data Type: Displays the type of data collected including topographic or bathymetric elevation, and seafloor feature object detection (e.g., sidescan).
- Primary Sensor Type: Indicates the type of technology used for collection, which provides an indication of spatial coverage within a survey area (e.g., multibeam echosounder versus single beam echosounder).
- Secondary Sensor Type: In the event that multiple sensors were deployed simultaneously during a survey, this provides an indication of coincident data available.
- Elevation Quality: (High, Medium, Low, None, and Unknown) A qualitative assessment of elevation data quality based on sensor type, acquisition or processing artifacts, and density of spatial coverage. Surveys where elevation data were not collected are coded as “None”, and surveys where no elevation data were available to evaluate are coded as “Unknown”.
- Intensity Quality: (High, Medium, Low, None, and Unknown) A qualitative assessment of intensity (i.e., multibeam backscatter, LiDAR (Light Detection and Ranging), reflectivity, or sidescan intensity) data quality based on usability to discern seafloor habitat types, acquisition or processing artifacts, density of spatial coverage, and degree of processing. Surveys where intensity data were not collected are coded as “None”, and surveys where no intensity data were available to evaluate are coded as “Unknown”.
- Data Time Period: Three time periods are displayed (2013–2003, 2002–1992, and earlier than 1992). More recent data collections are generally of better data quality given improvements in spatial positioning, resolution, and sensor quality. In addition, older datasets may not reflect the current condition of seafloor habitats in locations altered by disturbances.
- Ground-Truthing: This indicates the locations and types of ground-truthing that has been conducted within the study area.
- Habitat Map Product: Displays locations where benthic habitat maps have or have not been produced using survey data.

**Figure 4:**
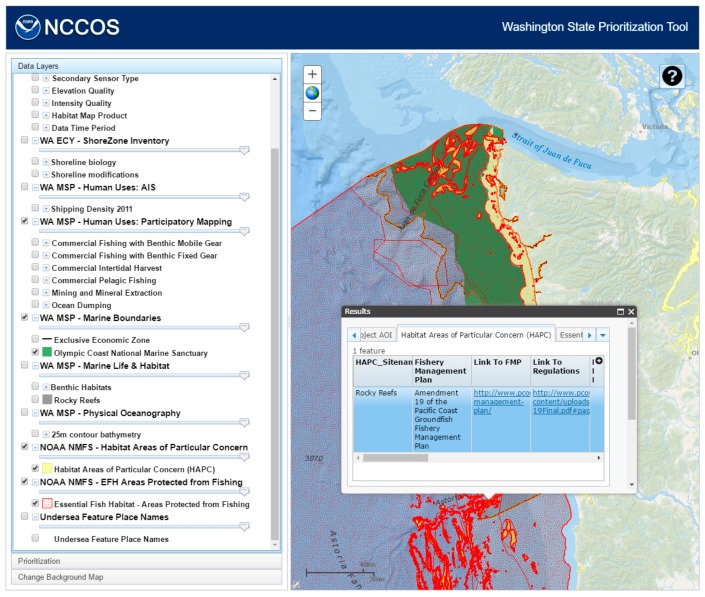
Compiled inventory of existing seafloor mapping data for Washington State made available to users through Washington State Prioritization Tool (WASP).

When the user clicks on the map, each layer that is turned on is queried. The features and attributes that are present at that point are shown in a popup window (Figure 4). When the user clicks on one of the rows in the tables, the corresponding feature will be highlighted on the map. This information will be used in the decision-making process in assigning priorities to the cells, such as selecting cells near the continental shelf edge as delineated by the 200 m bathymetry contour.

In the editing component, the invited respondent logged on to gain access to the tools to assign priorities, management issues, and ranking criteria (Selection Definitions—see below). Each respondent was given an account on NOAA’s GeoPortal, NOAA’s ArcGIS Online account. This is a GIS (Geographic Information System) application environment for use by NOAA employees, giving users the ability to share NOAA data, web maps, applications, tools, and web services with internal project teams as well as with NOAA partners and the public. The NOAA respondents were given the standard privileges, giving them the ability to create new content, share maps and apps, join and create groups, and edit features. All other external respondents were given a custom privilege, only allowing them to edit existing features. 

A polygon grid was created for the study area, which is defined by the Washington Marine Spatial Planning study area, covering the shoreline to the 1280 m (700 fathom) isobath. This dataset contains 996 cells, based on the Office of Coast Survey blocks of 4.8 km × 4.8 km. This grid was stored in a file geodatabase and contains fields for a unique grid number, priority, management issue, and three ranking criteria. The priority, management issue, and criteria fields were assigned attribute domains, which describe the valid values of the fields and enforce data integrity. The users were not permitted to add any custom text. The fields would accept only numeric values and the attribute domains translate these into defined text. The user was presented with the text descriptions of the selection choices and instructions on the choices. 

Selection Definitions: 

Priority: Select the priority (i.e., the relative measure of the need for seafloor mapping information) for a grid cell. The user must select one of the four options for each grid cell.

- Hig Priorityh—immediate need; of critical importance (may be required or mandated); the absence severely impacts services or decision-making. (e.g., “Need it now”).
- Medium—needed in the near future; non-critical importance but still of value; moderate impact on services or decision-making if not available. (e.g., “Need in the near future”).
- Low—undetermined future need; non-critical importance but still of value; no direct impact on services or decision-making if not available. (e.g., “Would be nice to have in the future”).
- None—Insufficient information to make a decision or not a priority for mapping.

Management Issue: Select the overarching management issue for a grid cell driving the “Priority” designation. While there can be multiple concerns, please select the single most critical issue.

- Living Resource Management—data needed to inform resource management decisions including harvested species as well as protected species and their habitats (e.g., Essential Fish Habitat (EFH), seabirds, marine mammals, fisheries, shellfisheries, aquaculture, submerged aquatic vegetation, etc.).
- Ecosystem Based Management—this includes better baseline information, proving oceanographic models.
- Safety and Navigation—information needed to support the management of maritime traffic or use activities.
- Coastal Inundation and Natural Coastal Hazards—information needed to support the management of areas at risk from coastal hazards and inundation.
- Spill Response—information needed to support spill response management or planning.
- Sediment Management—data needed to support dredging and management of sediment disposal areas, or sand mining.
- Cultural Heritage and Historical Resource—information needed to inform the management of locations of known cultural or historical significance.
- Marine Debris including Derelict Fishing Gear—information needed to inform the management of areas of marine debris convergence or impact.
- Defense and Homeland Security Activity Areas—information needed to inform areas with restrictive operational use.
- Other Regulatory—information needed to inform other permitting or regulatory assessments not captured by other categories (e.g., environmental assessments, National Environmental Policy Act, leasing, ownership, etc.).
- Research—information needed to inform research program investigations.
- Other—other management issue not included above.
- Insufficient Information—insufficient familiarity with location to be able to make a decision (associated with “None” priority).
- Not a Priority for Management—locations not a priority for management (associated with “None” priority).
- None—no Management Issues are associated with this cell.

Ranking Criteria (1 through 3): Select up to three Ranking Criteria options for each grid cell. The Ranking Criteria is intended to modify or describe the Management Issue in further detail. The Ranking Criteria are listed in descending order (1 being most important, 2 and 3 being successively less important.). The user must define at least one Ranking Criteria. The other two are optional.

- Multiple Use Conflict—an area with known, existing, multiple competing uses (e.g., commercial fishing and recreational boating).
- Managed Areas—special use, managed resource harvest areas, or other designated state/federal/tribal/local managed areas (e.g., EFH, shellfish beds, and dredge material disposal sites).
- Knowledge Gap—areas where there is no, limited, poor quality, or outdated information and where it is needed.
- Significant Natural Areas—areas known to be of unique or important ecological value, but not necessarily having any official or legislated designation (e.g., rocky intertidal, cold-water coral, kelp beds, etc.).
- High Use Areas—(e.g., ship traffic, fishing, and recreation).
- Existing Infrastructure—(e.g., jetties, cable, pipeline, etc.).
- Potential Infrastructure or other potential uses—area that could be targeted for future infrastructure projects or other new uses (e.g., cable, pipeline, wind/wave turbines, tidal energy devices, new dredge material sites, etc.).
- Other Important Areas—other activities not included above (e.g., research areas, cultural resources).
- None—not a priority for Management Issue.

**Figure 5:**
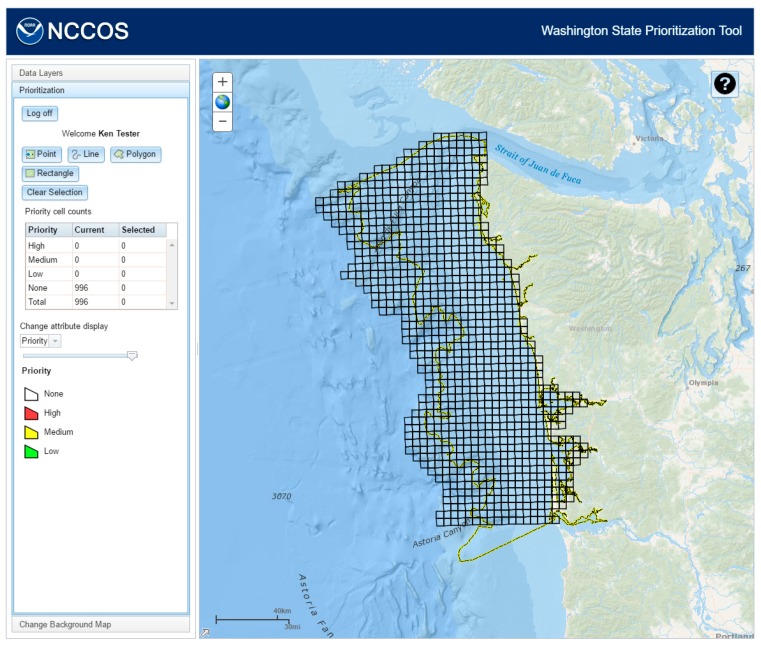
After logging into the NOAA GeoPortal, the user will have access to a feature service and editing tools.

For each respondent, a feature service was created in the NOAA GeoPortal using the polygon grid. The respondent was given the permission to edit the attributes, but not the geometry, of the feature service. Once this invitation was accepted, the respondent could log onto the prioritization website and edit their grid (Figure 5).

**Figure 6:**
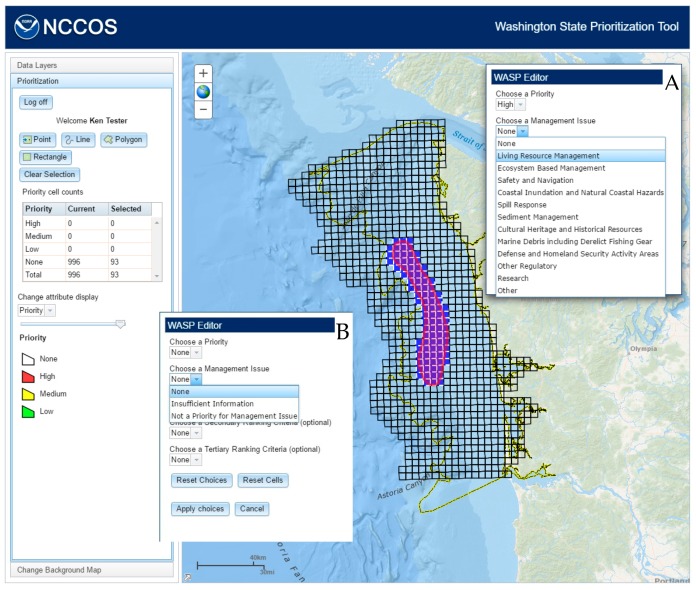
Each attribute has a set list of choices that the user must select (**A**). The list of available choices for management issue and ranking criteria will change depending on the chosen priority; e.g., if priority “None” is chosen (**B**).

**Figure 7:**
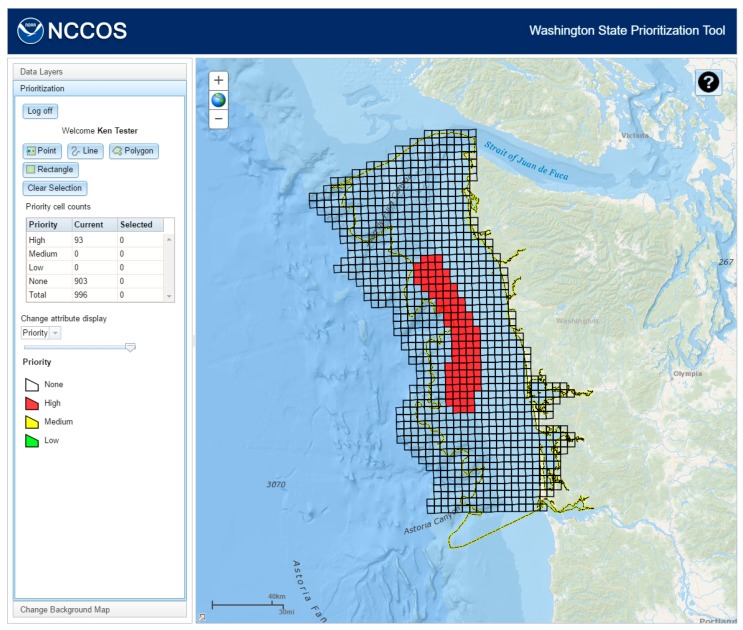
When the user saves the selection, the feature service will be updated and the cell counts table shows the new results.

The respondent can select features using the tools provided, selecting a single feature at a point or multiple features using a line, polygon, or rectangle. When features are selected, a window containing the drop-down attribute selections for priority, management issue, and ranking criteria is opened (Figure 6A). Depending on the priority chosen, the respondent will be presented with two different sets of choices for management issue and ranking criteria (Figure 6B). Choosing “None” will include management issues of “None”, “Insufficient Information”, and “Not a priority for Management Issue” and ranking criteria of “None” and “Knowledge Gap”. The other priorities will include all management issues except “Insufficient Information” and “Not a priority for Management Issue” and all ranking criteria. A management issue and at least one ranking criteria must be chosen before these edits can be saved by clicking the “Apply choices” button. If not, a warning dialog will appear listing the fields to be selected. The total number of cells to be designated as “high” or “medium” priority is limited to 300, 30% of the total, to force respondents to consider and weigh rankings. If the respondent selects more than that limit, a warning dialog will appear stating how many cells have been selected over that limit. The table (Priority cell counts) keeps track of how many cells have been selected and how many cells have been assigned the different priorities. The map will show the priority attribute by default, but the user can also display the management issue or ranking criteria attributes by selecting from a list in the “Change attribute display” section. Once the “Apply choices” button is clicked, the feature service will be updated with the new attributes (Figure 7).

Once the Prioritization exercise was closed, the editing permissions for all feature services were turned off. The respondent could still see their data, but could not make any further changes to the attributes. Each feature layer was exported into a file geodatabase on ArcGIS.com to maintain the attribute domains and downloaded to a local drive for analysis.

### 2.2. Spatial Analysis

#### 2.2.1. Chi-Square Test of Association in Responses

Participant survey data were exported from the participatory refinements into JMP (SAS Institute) to organize and analyze the user responses. Table 1 depicts the spatial prioritization submissions totaled across survey respondents, with the quantity of grid cells scored by priority (High, Medium, and Low) for each management issue category. This underlying contingency table provided insight towards the range in quantity of responses and the similarities among respondents in perceived needs and application. Furthermore, this tabular construct provided a statistical framework to test the association between row (issue) and column (priority) variables. The null hypothesis (H°) assumes that there is no association between the variables. As the survey data collected were nominal and resulting frequencies non-normally distributed, we used Pearson’s chi-square test statistic to assess whether observations were independent of each other [20,21]. Expected results and associated contingency values reflect what the responses might be in an idealized situation and are defined by:

Expected Value = the product of the corresponding row total and column total divided by the grand total (i.e., all responses in the survey) and where:

$$\text{Contingency Value}=\frac{{\left(\text{Observed Priority Value}-\text{Expected Priority Value}\right)}^{2}}{\text{Observed Priority Value}}$$

In addition to the observed priority values across Priority and Issue, Table 1 lists the percent that each cell count contributes to the grand total (total%), expected, and contingency calculations (cell Chi-square) for the management issues.

#### 2.2.2. Spatial Processing and Getis-Ord Gi* Hotspot Analysis

After gaining a deeper understanding of the relationships among Issues, Criteria, and Priorities using Chi-Square analysis, we conducted Getis-Ord Gi* Hotspot analysis to explore the spatial pattern and association of responses. With 14 possible management issues, eight possible selection criteria, and four levels of priority, there were 448 possible mapping permutations. Rather than map each of these permutations, we decided to map only those management issues that were determined to be significantly higher than expected in the “High Priority” classification and/or those where the total issue response exceeded 10% of the grand total. As such, the following six management issues were mapped: Ecosystem Based Management, Living Resource Management, Coastal Inundation and Natural Coastal Hazards, Other Regulatory, Sediment Management, and Research. 

The Esri ArcGIS Geostatistical Hot Spot Analysis tool was used to process the responses for these six issues and determine if statistically significant clusters or patterns of values exist that would more definitively represent areas to prioritize [22,23,24]. At a basic level, the tool works by looking at each grid cell within a context of neighboring cells. A cell with a high score may be interesting, but to be statistically significant, it would need a high score and be surrounded by other cells with high scores.

The process returns a statistic (z-score)—in essence, a standard deviation value—for each feature in the dataset. For statistically significant positive z-scores, a larger z-score is indicative of intense clustering of high values. Conversely, statistically significant negative z-scores are indicative of intense clustering of low values. The tool also provides a probability statistic (*p*-value) that measures whether a spatial pattern reflects random chance. In areas with appropriately small *p*-values and either a very high or a very low z-score, it is unlikely that the spatial pattern is completely random and thus is a significant cluster.

#### 2.2.3. Response Integration and Spatial Prioritization

To identify regions in the area of interest as initial priority mapping targets, results of the six hotspot analyses were integrated into a single map depicting the cumulative frequency of hotspot detection. Furthermore, the original z-scores from individual hotspot maps were modeled using ESRI’s geostatistical analyst “kriging” tool. Kriging is an interpolation technique in which the surrounding measured values are weighted to derive a predicted value for an unmeasured location [25,26]. Weights are based on the distance between the measured points, the prediction locations, and the overall spatial arrangement among the measured points. Kriging is unique among the interpolation methods in that it provides an easy method for characterizing the variance, or the precision, of predictions. This resulted in an interpolated hotspot map (heat map) for each of the six priority management issues identified. The heat maps were then integrated using ESRI’s map algebra tool to generate a composite heat map. The resulting heat map was designed as a visual queue to initiate subsequent discussion and modifications by the respondents during the participatory refinement workshop (Section 2.3). As such, the heat map was not designed to define the ultimate priority areas; rather, it was designed to provide a preliminary basis so as to invigorate further refinements during the participatory workshop.

### 2.3. Participatory Refinements

A second spatial prioritization planning workshop was held 14 May 2015 in Lacey, WA to share the results of the spatial analysis with the group and seek refinements. Workshop participants were divided into two sub-groups (i.e., Inshore and Offshore), based on interest, to discuss and comment on the proposed priority areas identified through the analysis. Discussions focused on the need to expand, contract, or add additional areas of management significance not captured through the spatial prioritization exercise. The group focused on reviewing supporting information available within the priority areas to ascertain whether sufficient existing seafloor mapping data was available which would preclude the necessity for additional collection. Several locations were identified meeting this criterion, and through participatory refinements, boundary modifications were proposed and comments captured. A total of sixteen minor boundary modifications were annotated that were incorporated into the subsequent final analysis and revisions.

## 3. Results

### 3.1. Chi-Square Test of Association in Responses

Chi-square values in Table 1 greater than 54.6 allow us to reject the null hypothesis and confirm there is a statistically significant association between Management Issues and Priority beyond random chance. These significant values are shaded as light green if the value was significantly higher than expected, and as light red if significantly lower than expected (see Section 2.2.1). Additionally, cells shaded in dark green indicate where a management issue received a total number of responses that exceeded 10% of the grand total. We conclude, therefore:

(a) Cell chi-square values for Living Resource Management, Coastal Inundation and Natural Coastal Hazards; Other Regulatory; Sediment Management; and Research suggest respondents implicitly considered them to be a high priority.
(b) The Issue of Ecosystem Based Management was the most often cited management issue across respondents; however; the cell chi-square value was not significant at the “High Priority” level.
(c) Living Resource Management and Coastal Inundation and Natural Coastal Hazards both exceeded 10% of the overall responses and were a selected as a high priority more often than otherwise expected.
(d) “No Response” was the most frequent occurrence in the survey, representing 32% of the grand total.
(e) Marine Debris was the least frequently selected management issue in the survey, representing 0.8% of all responses.

Additional chi-square tests were performed to determine if relationships also exist between Management Issues and Ranking Criteria and the results are summarized in Table 2.

### 3.2. Spatial Processing and Getis-Ord Gi* Hotspot Analysis

**Figure 8:**
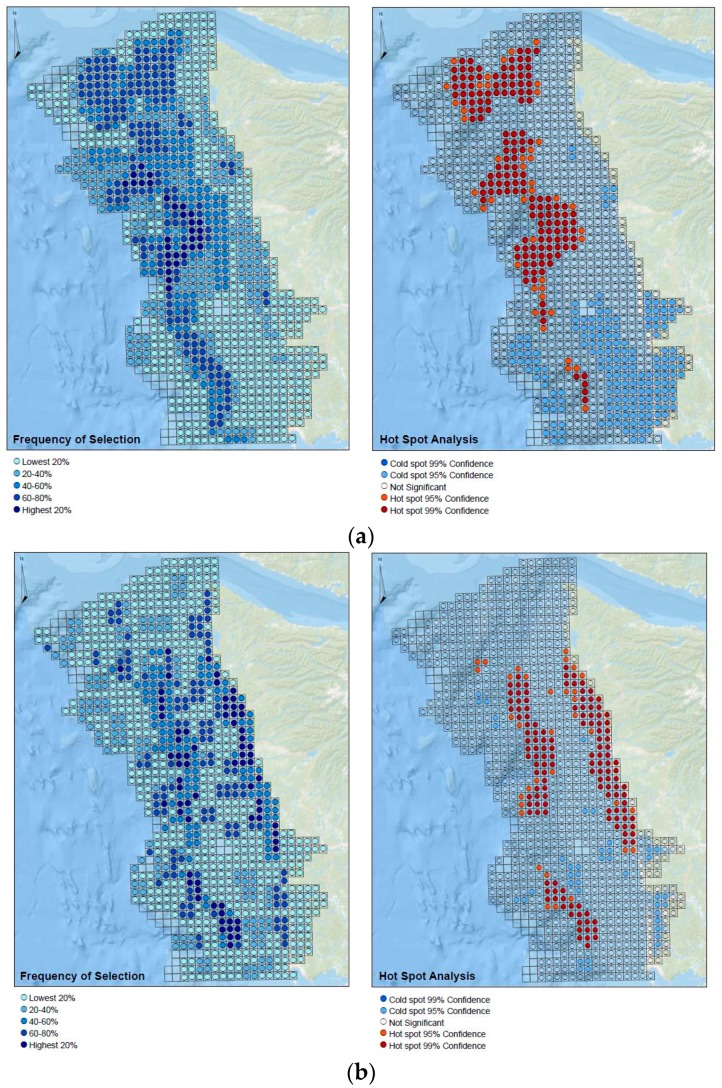

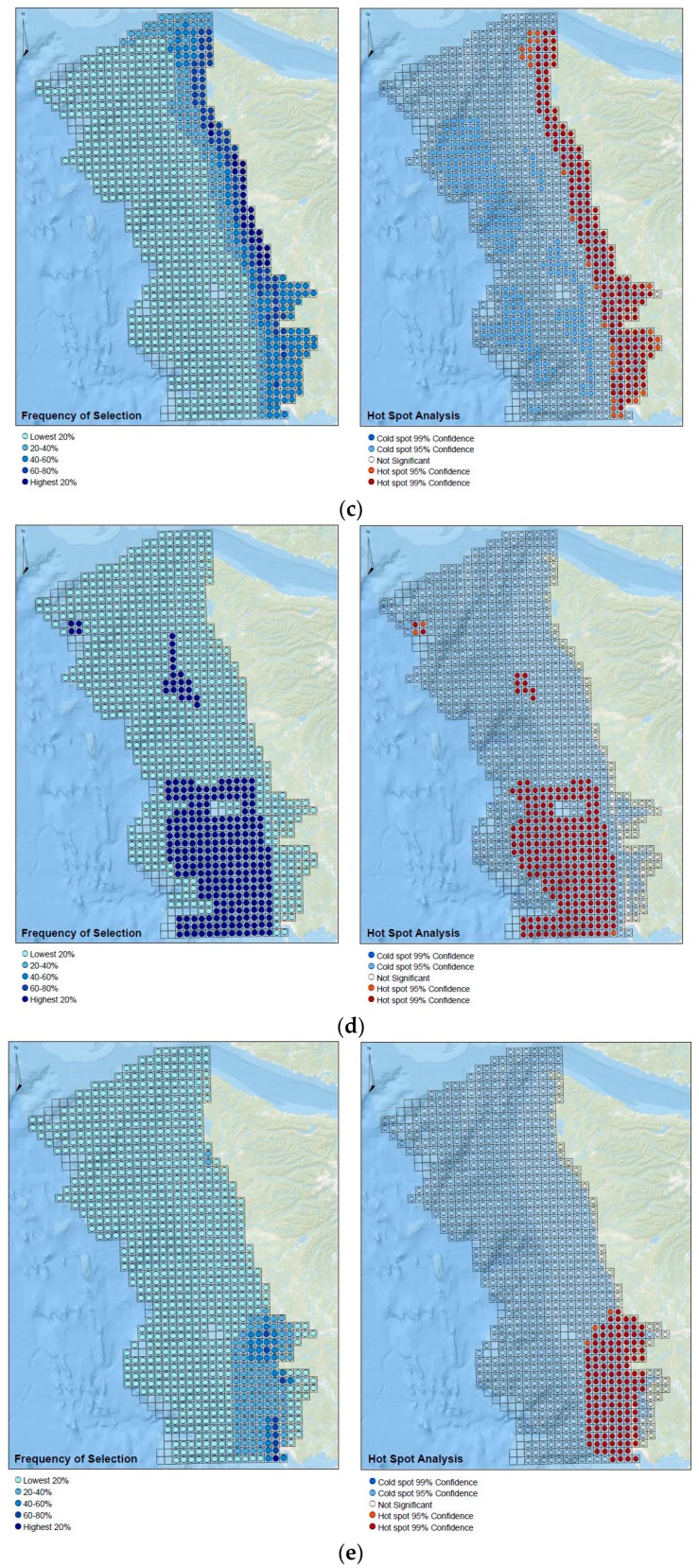

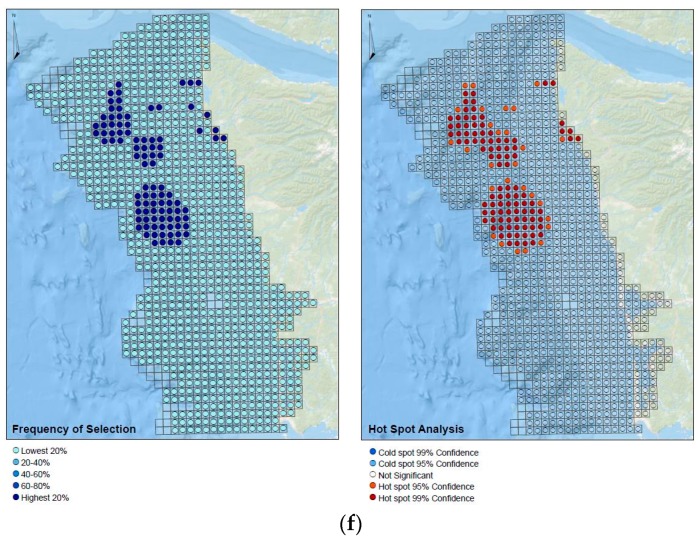
(**a**) Frequency of “high priority” selections and associated hotspots for Living Resource Management; (**b**) frequency of “high priority” selections and associated hotspots for Ecosystem-based Management; (**c**) frequency of “high priority” selections and associated hotspots for Coastal Inundation and Natural Coastal Hazards; (**d**) frequency of “high priority” selections and associated hotspots for Other Regulatory; (**e**) frequency of “high priority” selections and associated hotspots for Sediment Management; and (**f**) frequency of “high priority” selections and associated hotspots for Research.

Figure 8a–f presents maps of the frequency of “high priority” selections tallied across all respondents for the six significant management issues identified (left panel) alongside the associated Getis-Ord Gi* hotspot analyses. Frequency of selection analysis is classified into 20 percentile groupings, and Getis-Ord Gi* analyses are mapped as “hot spots” (red) or cold spots (blue) with associated statistical confidence. Where the z-score was not statistically significant, cells are transparent. 

**Figure 9:**
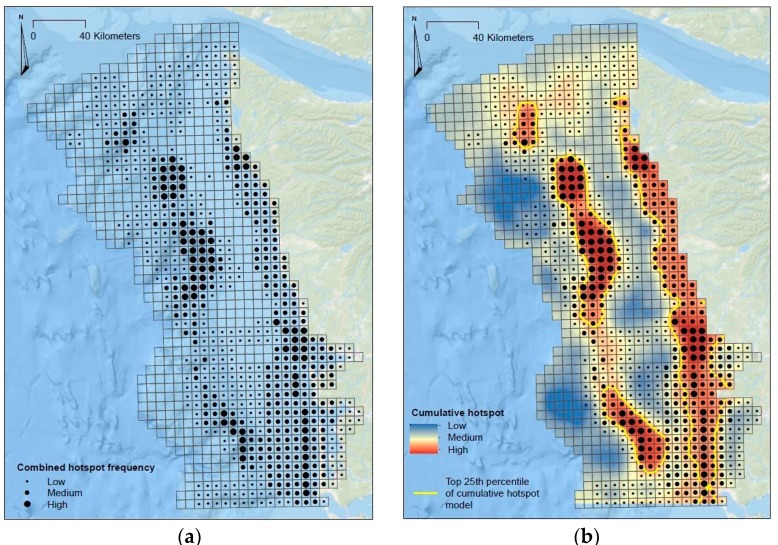
Frequency of hotspot analysis (**a**); and composite heat map (**b**).

To identify regions in the area of interest as initial priority mapping targets, the six hotspot analyses were integrated into a single map depicting the cumulative frequency of hotspot detection. The heat maps were then integrated using ESRI’s map algebra tool to generate a composite heat map and subsequently interpolated using kriging to derive predicted values between hotspots. As a starting point to identify priority mapping targets, we generated an isopleth around the top 25th percentile of the composite heat map (Figure 9).

### 3.3. Response Integration and Spatial Prioritization

**Figure 10:**
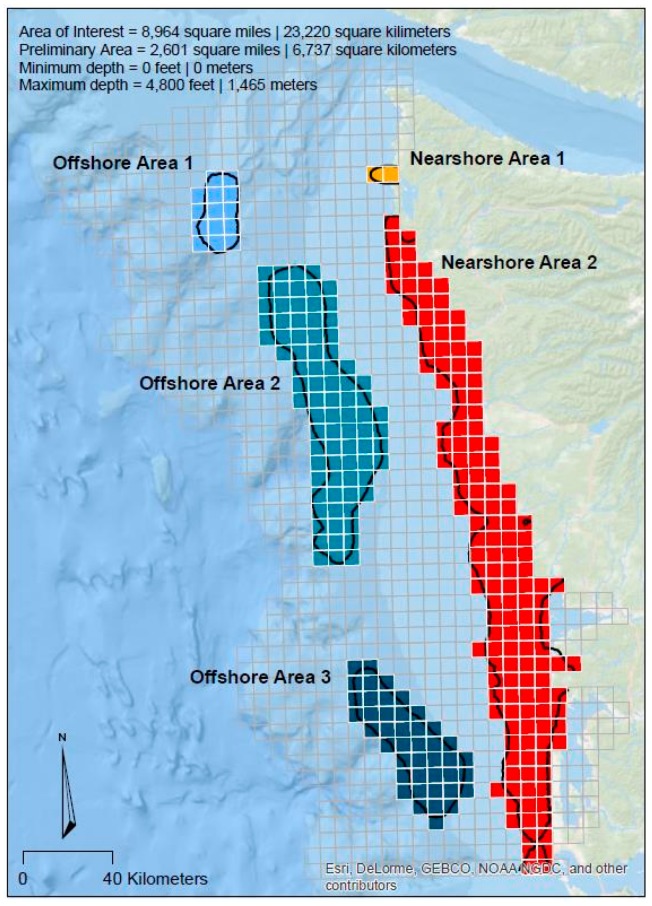
Preliminary priority mapping areas identified through cumulative hotspot analysis.

A total of five unique regions, three offshore and two nearshore, were identified using kriging (Figure 10). By comparing the priority boundaries and the gridded versions of the survey responses, a clearer perspective can be gained on the issues and criteria that prevailed in these areas, and where future mapping and analysis should be targeted. Noteworthy statistics for each preliminary mapping area include:

Offshore area 1: Total area = 67 km^2^; minimum depth = 110 m; maximum depth = 740 m; represents 1.5% of the entire area of interest, represents 2% of all “high priority” selections made by survey respondents; captures 4% of all “high priority” selections for the “living resource management” issue. 

Offshore area 2: Total area = 1911 km^2^; minimum depth = 84 m; maximum depth = 1463 m; represents 8% of the entire area of interest, represents 13% of all “high priority” selections made by survey respondents; captures 20% of all “high priority” selections for the “research” issue. 

Offshore area 3: Total area = 1002 km^2^; minimum depth = 84 m; maximum depth = 1026 m; represents 4% of the entire area of interest, represents 6% of all “high priority” selections made by survey respondents; captures 17% of all “high priority” selections for the “other regulatory” issue. 

Nearshore area 1: Total area = 47 km^2^; minimum depth = 0 m; maximum depth = 43 m; represents 0.2% of the entire area of interest, represents 0.6% of all “high priority” selections made by survey respondents.

Nearshore area 2: Total area = 3450 km^2^; minimum depth = 0 m; maximum depth = 73 m; represents 15% of the entire area of interest, represents 27% of all “high priority” selections made by survey respondents; captures 23% of all “high priority” selections for the “ecosystem based management” issue; captures 52% of all “high priority” selections for the “coastal inundation and hazards” issue, captures 67% of all “high priority” selections for the “sediment management” issue. 

Additional summary statistics of response attributes for each of these areas is provided in Table 3a–e. Selection criteria associated with each management issue that were statistically significant are bolded and italicized. 

## 4. Discussion and Conclusions

Over the last decade, the importance of the U.S. ocean-based economy has continued to increase despite declining investment in activities to support this economic sector. For example, in 2013, employment within the U.S. ocean economy grew by three percent, almost twice as fast as the U.S. economy as a whole and accounted for $117 billion in wages [27]. However, investment by the federal government in ocean science, protection, and management activities has been decreasing. From fiscal years 2012 to 2016, federal ocean related expenditures decreased from $11.7 to $10.8 (billions) [2]. While investment in ocean related activities may remain constant in the near-term, decision-making tools that increase the efficiency of ocean planning and mapping will help focus where to best direct future efforts and finite resources.

Furthering progress to map U.S. ocean and coastal waters has been challenged by the enormous extent of remaining unmapped areas and the absence of a national mapping strategy [5]. Moreover, even in regions where mapping has been completed, coverage reflects a disparate patchwork of survey quality, data resolution, legacy, and public accessibility. For example, a gap analysis of existing mapping data within southern California found that 87% of the region remained unmapped [19]. To overcome these challenges, many coastal states with legislative mandates to conduct ocean planning have taken the initiative to develop their own state-wide mapping strategies [12,17,18]. 

Washington State embodies many of the aforementioned characteristics including a strong ocean economy, a legislated ocean planning process, large unmapped areas, and the need to implement a process to identify future mapping priorities [12]. Consequently, the prioritization process conducted in Washington resulted in a map, based on participant input, which identified shared priorities and needs for subsequent mapping efforts. It is important to note, however, that the results of this process were driven entirely by independent inputs from the regional agencies and coastal mapping community members who chose to participate. It is entirely possible that if one or more additional agencies had chosen to participate (or alternately contributors chose not to), the resulting integrated priority areas may have shifted. The intent of this effort was to provide a tool and companion process to “socially engineer” differing priorities through engagement activities, not to test the sensitivity of such a process. As such, we have not provided a specific sensitivity analysis using statistical resampling techniques. Moreover, the five priority areas (Figure 10) do not reflect every “high” priority cell identified in Figure 8a–f, an inherent consequence of the hotspot analysis and statistical convergence techniques employed. As a precaution when using the techniques proposed, the user should be aware that small clusters of “high” priority cells are still of importance, and perhaps warrant mapping, but were sufficiently, spatially isolated enough so as not be included in the five larger priority clusters. 

Specifically, the outcome of this process provided key components needed to garner broad, regional support and strategize future seafloor mapping efforts. These outcomes included: (1) the identification of five discrete priority locations; (2) the explicit identification of the management application of seafloor mapping data within the study area; and (3) the identification of informational products most needed by users. 

Based on input from participants in Washington, “seafloor topography and texture” were selected as the most important informational products applicable for use within the study area. These products are instrumental for depicting the shape, depth, texture, roughness, and composition of the seafloor [28]. High resolution ship-based acoustic sonar systems (e.g., multibeam echosounders) and airborne bathymetric LIDAR (Light Detection and Ranging) [5] were identified as the most applicable remote sensing systems able to generate accurate digital elevation models [29] and seafloor intensity mosaics [30], products highly desired for use and application in this region. 

Specific locations identified during the Washington State process enabled detailed planning for identifying the optimum remote sensing systems and project costs, estimating survey time and assessing ship time necessary to complete the priority areas. Consequently, 21 operational days (14 in 2016 and 7 in 2017) were secured to map the three offshore priority area using the NOAA ship *Rainier *(S221). The optimum sonar systems were selected to best address project area conditions and the observations needed (e.g., water depths, seafloor relief, water-column imaging); survey line spacing and orientation were planned to meet the identified requirements; and bathymetry, topography, acoustic backscatter, and water-column products were produced to fulfill user needs and applications. The results of the Washington State prioritization were instrumental in providing clearly defined locations and applications for mapping data and solidifying cohesive support across multiple agencies to secure the needed ship time. In the past, the absence of clearly defined locations and collaboration has failed to garner the mapping support needed in this region.

Federal and state strategic planning is more effective when requirements are known and clearly articulated and direct application of investments can be demonstrated. If conducted prudently and strategically, the investment in seafloor mapping will provide valuable return benefits, by promoting the availability of more complete information to aide in better planning and management of coastal resources. The seafloor mapping prioritization conducted in Washington State provides a successful process that can be utilized by other states and regions to aid and inform ocean mapping and decision-making. 

## Figures and Tables

**Table 1:** Spatial prioritization submissions totaled across survey respondents.

	None	Low	Med	High	S	Primary Criteria		None	Low	Med	High	S	Primary Criteria
**No Response Given**						N/A	**Safety and Navigation**						Multiple use conflict
Count	4408	0	0	0	4408		Count	0	360	60	61	481	High use areas
Total %	32.0	0.0	0.0	0.0	32.0		Total %	0.0	2.6	0.4	0.4	3.5	
Expected	1469.9	1161.0	882.3	894.8			Expected	160.4	126.7	96.3	97.6		
Cell Chi^2	5873.1	1161.0	882.3	894.8			Cell Chi^2	160.4	429.6	13.7	13.7		
**Ecosystem Based Management**						Managed areas	**Other**						Other important areas
Count	0	1401	1123	846	3370	Knowledge gap	Count	0	382	0	0	382	
Total %	0.0	10.2	8.2	6.1	24.5	Significant natural areas	Total %	0.0	2.8	0.0	0.0	2.8	
Expected	1123.7	887.6	674.5	684.1			Expected	127.4	100.6	76.5	77.5		
Cell Chi^2	1123.7	296.9	298.2	38.3			Cell Chi^2	127.4	786.9	76.5	77.5		
**Living Resource Management**						Potential infrastructure	**Spill Response**						Significant natural areas
Count	0	53	772	877	1702	Knowledge gap	Count	0	256	76	13	345	
Total %	0.0	0.4	5.6	6.4	12.4	Significant natural areas	Total %	0.0	1.9	0.6	0.1	2.5	
Expected	567.5	448.3	340.7	345.5		Other important areas	Expected	115.0	90.9	69.1	70.0		
Cell Chi^2	567.5	348.6	546.1	817.7			Cell Chi^2	115.0	300.1	0.7	46.4		
**Coastal Inundation and Natural Coastal Hazards**						Existing infrastructure	**Defense and Homeland Security**						Other important areas
Count	0	786	322	470	1578	Other important areas	Count	0	269	0	0	269	
Total %	0.0	5.7	2.3	3.4	11.5		Total %	0.0	2.0	0.0	0.0	2.0	
Expected	526.2	415.6	315.9	320.3			Expected	89.7	70.9	53.8	54.6		
Cell Chi^2	526.2	330.0	0.1	69.9			Cell Chi^2	89.7	554.1	53.8	54.6		
**Other Regulatory**						Potential infrastructure	**Not a Priority for Management**						None
Count	0	0	260	259	519		Count	132	0	0	0	132	
Total %	0.0	0.0	1.9	1.9	3.8		Total %	1.0	0.0	0.0	0.0	1.0	
Expected	173.1	136.7	103.9	105.4			Expected	44.0	34.8	26.4	26.8		
Cell Chi^2	173.1	136.7	234.6	224.1			Cell Chi^2	175.9	34.8	26.4	26.8		
**Sediment Management**						Knowledge gap	**Marine Debris**						Managed areas
Count	0	9	31	176	216		Count	0	112	0	0	112	
Total %	0.0	0.1	0.2	1.3	1.6		Total %	0.0	0.8	0.0	0.0	0.8	
Expected	72.0	56.9	43.2	43.8			Expected	37.3	29.5	22.4	22.7		
Cell Chi^2	72.0	40.3	3.5	398.3			Cell Chi^2	37.3	230.7	22.4	22.7		
**Research**						Knowledge gap	**Insufficient Information**						N/A
Count	0	0	113	94	207		Count	53	0	0	0	53	
Total %	0.0	0.0	0.8	0.7	1.5		Total %	0.4	0.0	0.0	0.0	0.4	
Expected	69.0	54.5	41.4	42.0			Expected	17.7	14.0	10.6	10.8		
Cell Chi^2	69.0	54.5	123.6	64.3			Cell Chi^2	70.6	14.0	10.6	10.8		

Pink: significantly ***less*** than expected; light green: significantly ***more*** than expected; dark green: >10% of all responses.

**Table 2:** Primary selection criteria that were determined to be significantly associated with management issue.

Management Issue	Significant Primary Criteria
Ecosystem Based Management	Managed Areas
	Knowledge Gap
	Significant Natural Areas
Living Resource Management	Potential Infrastructure
	Knowledge Gap
	Significant Natural Areas
	Other Important Areas
Coastal Inundation and Coastal Hazards	Existing Infrastructure
	Other Important Areas
Other Regulatory	Potential Infrastructure
Sediment Management	Knowledge Gap
Research	Knowledge Gap
Other	Other Important Areas
Spill Response	Significant Natural Areas
Defense and Homeland Security	Other Important Areas
Not a Priority for Management	None
Marine Debris	Managed Areas

**Table:** (**a**)

Issue	# Responses	% of Responses	Listed Criteria Captured
Ecosystem based management	62	34.6%	Multiple use, ***managed areas, knowledge gap, significant natural areas***, potential infrastructure
Living resource management	51	28.5%	***Knowledge gap, Significant natural area***
Coastal inundation	28	15.6%	***Other important areas***
Safety and Navigation	14	7.8%	***Multiple use***
Other	13	7.3%	***Other important areas***
Research	10	5.6%	***Knowledge gap***
Other regulatory	1	0.6%	***Potential infrastructure***
***TOTALS***	***179***	***100.0%***	

**Table:** (**b**)

Issue	# Responses	% of Responses	Listed Criteria Captured
Living resource management	355	29.8%	***Knowledge gap, significant natural area,*** existing infrastructure
Ecosystem based management	280	23.5%	Multiple use, ***managed areas, knowledge gap, significant natural areas***, high use areas
Coastal inundation	216	18.1%	Significant natural areas
Safety and Navigation	82	6.9%	***Multiple use***
Spill response	70	5.9%	***Significant natural areas***
Defense & homeland security	54	4.5%	***Other important areas***
Other	54	4.5%	***Other important areas***
Research	42	3.5%	***Knowledge gap***
Other regulatory	39	3.3%	***Potential infrastructure***, other important areas
***TOTALS***	***1192***	***100.0%***	

**Table:** (**c**)

Issue	# Responses	% of Responses	Listed Criteria Captured
Ecosystem based management	228	41.61%	Multiple use, ***knowledge gap, significant natural area***, high use area, potential infrastructure
Coastal inundation	118	21.53%	Significant natural areas, potential infrastructure, ***other important areas***
Living resource management	109	19.89%	***Knowledge gap, significant natural area***
Safety and Navigation	45	8.21%	***Multiple use, high use area***
Other regulatory	43	7.85%	***Potential infrastructure***
Sediment management	3	0.55%	***Knowledge gap***
Research	2	0.36%	***Knowledge gap***
***TOTALS***	***548***	***100.0%***	

**Table:** (**d**)

Issue	# Responses	% of Responses	Listed Criteria Captured
Coastal inundation	6	27.3%	Managed areas, knowledge gap, potential infrastructure, ***other important areas***
Ecosystem based management	5	22.7%	***Managed areas, knowledge gap***
Living resource management	3	13.6%	***Knowledge gap, significant natural area***
Safety and Navigation	2	9.1%	***Multiple use conflict***
Spill response	2	9.1%	***Significant natural areas***
Research	2	9.1%	***Knowledge gap***
Other	2	9.1%	***Other important areas***
***TOTALS***	22	100.0%	

**Table:** (**e**)

Issue	# Responses	% of Responses	Listed Criteria Captured
Living resource management	488	23.4%	Managed areas, ***knowledge gap, significant natural areas***, high use areas
Coastal inundation	482	23.1%	Managed areas, knowledge gap, significant natural areas, high use areas, ***existing infrastructure***, potential infrastructure, ***other important areas***
Ecosystem based management	420	20.2%	Multiple use, ***managed areas, knowledge gap, significant natural area***, potential infrastructure
Safety and navigation	253	12.1%	***Multiple use***, managed areas, ***high use areas***, existing infrastructure, potential infrastructure
Sediment management	121	5.8%	Multiple use, managed areas, ***knowledge gap***, significant natural area
Spill response	95	4.6%	***Significant natural areas***, high use areas, existing infrastructure
Other	67	3.2%	***Other important areas***
Other regulatory	59	2.8%	***Potential infrastructure***
Marine debris	34	1.6%	***Managed areas***
Defense and homeland security	34	1.6%	***Other important areas***
Research	30	1.4%	Managed areas, ***knowledge gap***
***TOTALS***	2083	100.0%	
